# Supplementary material for: Verifying molecular clusters by 2-color localization microscopy and significance testing
Source: Sci Rep. 2020 Mar 6;10:4230. doi: 10.1038/s41598-020-60976-6 (PMC7060173; doi:10.1038/s41598-020-60976-6)
Supplement: Supplementary file 2 — Supplementary Information2. [file 41598_2020_60976_MOESM2_ESM.zip › 2-CLASTA-master/2-CLASTA_plugin_manual.pdf]

Manual

ImageJ plugin
2-CLASTA

by Christoph Hüsson

Andreas M. Arnold[1], Magdalena C. Schneider[1], Christoph Hüsson[2], Robert Sablatnig[2], Mario Brameshuber[1], Florian Baumgart[1], Gerhard J. Schütz[1]

[1] Institute of Applied Physics, TU Wien
[2] Institute of Visual Computing & Human-Centered Technology, TU Wien

Version 1.0

Contents

1 About **1**
1.1 Installation 1
1.2 Compatibility 1

2 Running an analysis **2**
2.1 Parameters-window 2
2.2 Selection of regions of interest 5
2.3 Results-window & -export 6

1 About

The plugin 2-CLASTA provides a method for analysis of two-color single-molecule microscopy localization data with the **2-C**olor **L**ocalization microscopy **A**nd **S**ignificance **T**esting **A**pproach described in the corresponding paper [1].

1.1 Installation

Please follow the instructions of your respective ImageJ-Distribution for installing plugins. Generally, it should be sufficient to place the "2-CLASTA_Analysis.jar"-file in the plugins\-folder of your ImageJ-installation.
Alternatively, install 2-CLASTA using the ImageJ-menu: 'Plugins' $\longmapsto$ 'Install PlugIn...' and then select the "2-CLASTA_Analysis.jar"-file. It may be necessary to restart ImageJ afterwards.

1.2 Compatibility

2-CLASTA requires JRE 8 to be installed (download). It is recommended to use a recent version, especially in regard to the latest performance-optimizations for chart-rendering.

2-CLASTA has been tested on

- Windows 10, 64-bit
- Windows 10, 32-bit
- MacOS
- Linux

and works with

- ImageJ 2
- Fiji (ImageJ 1.52n)

2 Running an analysis

In ImageJ, **Plugins** ⟼ **2-CLASTA** ⟼ **Run 2-CLASTA analysis** starts a new analysis.

2.1 Parameters-window

The parameter-window will open, allowing users to select **Datasources** and define **Parameters**.

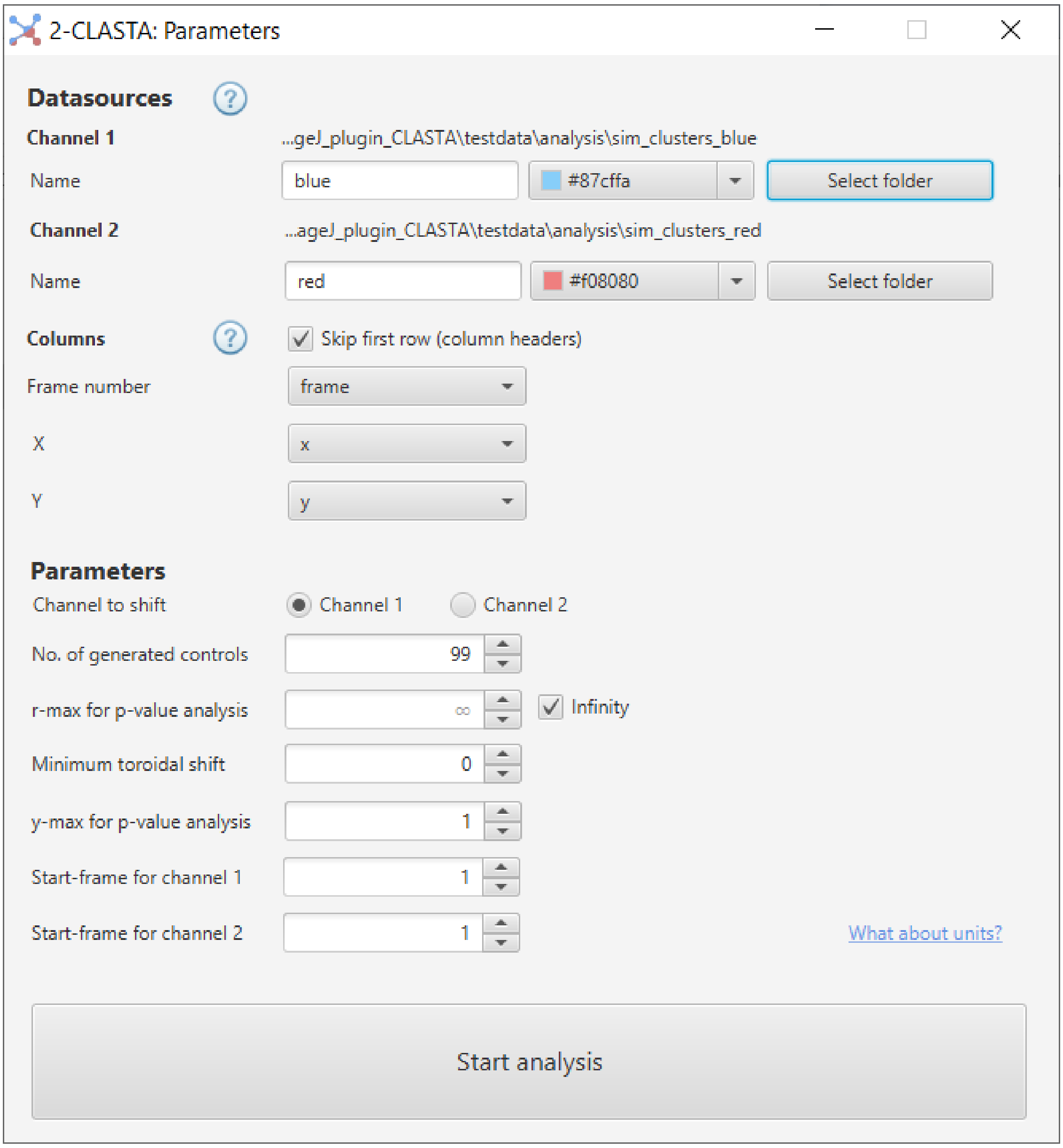

Figure 1: The parameters-window for a new 2-CLASTA-analysis. The selected datasources will be persisted for each user.

Datasources

Each of the two captured colors is considered as one **Channel**. To help identify these channels, users may **Name** them freely with up to *30 characters* each and choose appropriate **Colors** which will be used for displaying the channels throughout the process. We recommend the use of easily distinguishable colors. Please also bear in mind that the results might be shared with people that suffer from *Dyschromatopsia*, who often are challenged by distinguishing red and green.

2-CLASTA accounts for multiple experiments being analysed in one batch and thus asks for **one folder** per channel. The **Select folder**-buttons will open a dialogue to choose a directory containing the *Csv-files* of the respective channel.

IMPORTANT: THE NUMBER OF FILES PER CHANNEL AND THEIR STRUCTURE (E.G. NUMBER OF COLUMNS, COLUMN-ORDER, PRESENCE OF COLUMN-HEADERS) NEED TO BE IDENTICAL! FILES IN EACH FOLDER ARE SORTED ALPHA-NUMERICALLY AND TWO FILES ARE MAPPED TO EACH OTHER WHEN THEIR POSITION IN THIS SORTED LIST IS IDENTICAL. TWO SUCH FILES ARE CONSIDERED A *Fileset*.

After selecting a folder, its path will be shown next to the channel's label. The first Csv-file in this folder will be analysed to identify the column's headers. In case no headers are present, users should untick the **Skip first row (column headers)**-checkbox. If headers are present, the columns will be listed in each dropdown-list of the **Columns**-section. The columns are assigned in the order of their occurrence in the Csv-files:

1. frame number (optional)
2. x-coordinate
3. y-coordinate

If this structure is not met by the selected files, users need to change the column-mapping accordingly by selecting the appropriate header-name from each column's dropdown-list. This also applies for Csv-files lacking a header-row - their columns will then instead be enumerated (starting at 1) in the dropdown-lists.

IMPORTANT: ALL VALUES ARE ASSUMED TO BE PROVIDED IN UNITS OF THE MEASUREMENT AND WILL BE PROCESSED WITHOUT CONVERSION! THIS ALSO HOLDS TRUE FOR THE PARAMETERS THAT WILL BE INTRODUCED BELOW.

Parameters

Once the datasource has been selected, it is necessary to choose suitable parameters for the analysis.

PLEASE NOTE: THE SAME PARAMETERS WILL BE APPLIED FOR ALL SELECTED FILE-SETS!

Channel to shift determines on which channel a toroidal shift [2] will be applied. The localizations of the other channel will remain fixed at their original positions.
DEFAULT: CHANNEL 1

No. of generated controls is the number of randomized toroidal shifts that will be applied to each fileset. A higher number of controls results in higher resource-consumption but higher stability of the calculated p-value.
DEFAULT: 99

r-max for p-value analysis defines the maximum nearest neighbour distance between the localizations of both channels to take into account during analysis. Distances higher than this value will not to be considered [1].
DEFAULT: ∞

Minimum toroidal shift. The components of the toroidal shift vector $\vec{v}$ are chosen randomly in the interval $[v_{min}, roi - v_{min}]$, where v_{min} is the minimum shift and roi the extension of the region of interest in the respective coordinate.
DEFAULT: 0

y-max for p-value analysis defines a cut-off for calculating and displaying the CDF (cumulative distribution function) of nearest-neighbour-distances on the y-scale. The information-content diminishes for probability-values approaching 1.0. Restricting this value saves resources. However, this cut-off is only applied if it does not prevent the CDF's x-axis from reaching r-max. This parameter has no effect on the resulting p-value.
DEFAULT: 1

Start-frame for channel 1 & 2 allows to omit all data prior to the selected start frame (can be chosen independently).
DEFAULT: 1

After all parameters have been selected, the user will be taken to the next step by clicking the **Select region of interest**-button.

2.2 Selection of regions of interest

The following step needs to be repeated for each fileset.

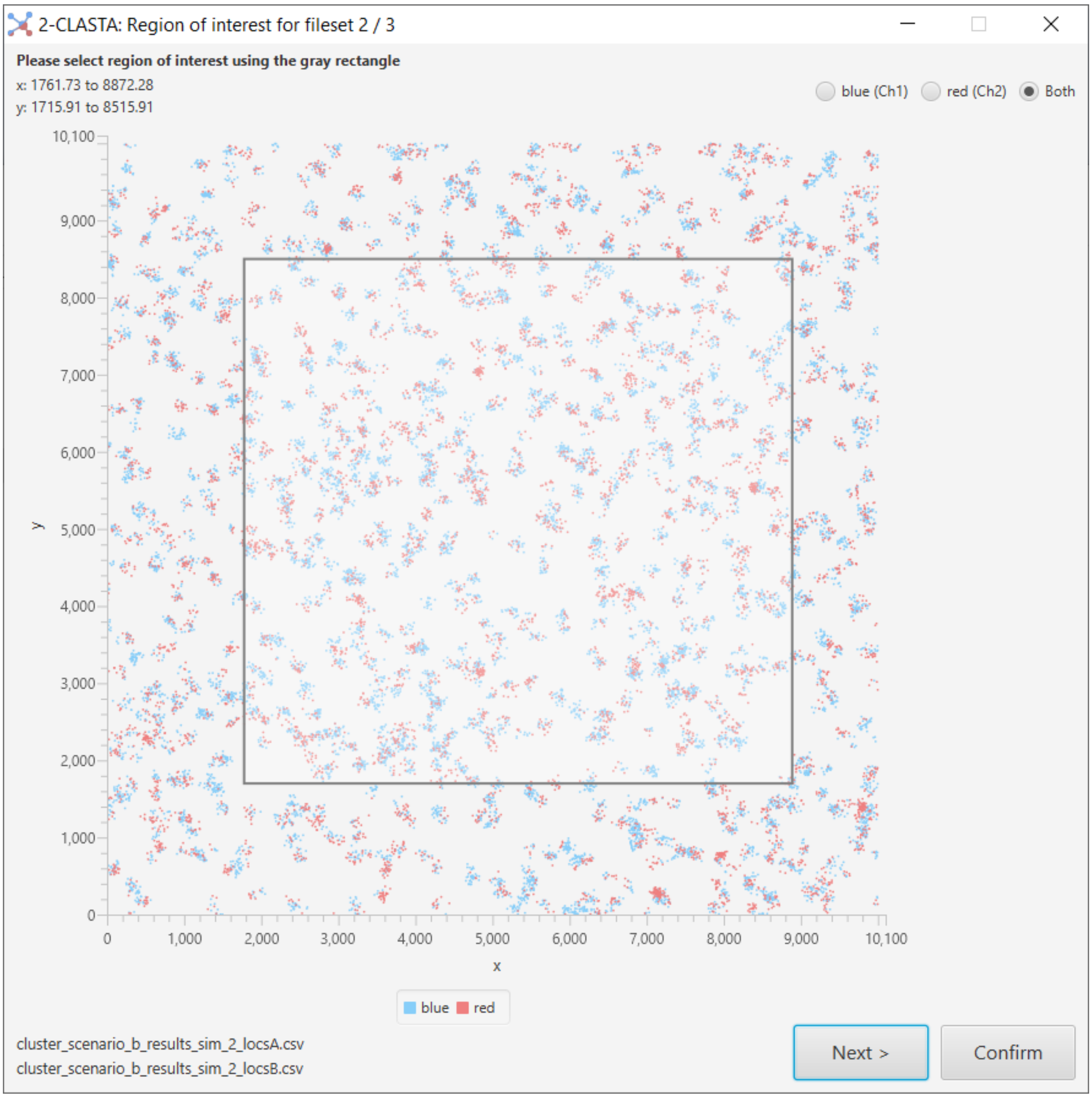

Figure 2: The *Region of interest-selection*-window for the second of three filesets. The user selects the region of interest by dragging and resizing the gray rectangle.

An overlay of both channels will be shown initially. Users can display each channel individually using the radio-buttons in the upper right corner.

After the region of interest has been selected, the user can either go to the **Next** fileset or press the **Confirm**-button to immediately run the analysis on the prepared filesets. Note that the current fileset's index as well as the total number of filesets to process is shown in the window-title.

2.3 Results-window & -export

When the regions of interest have been confirmed, the results-window will open to present the analysis-details. The obtained results are shown in a four-tile view.

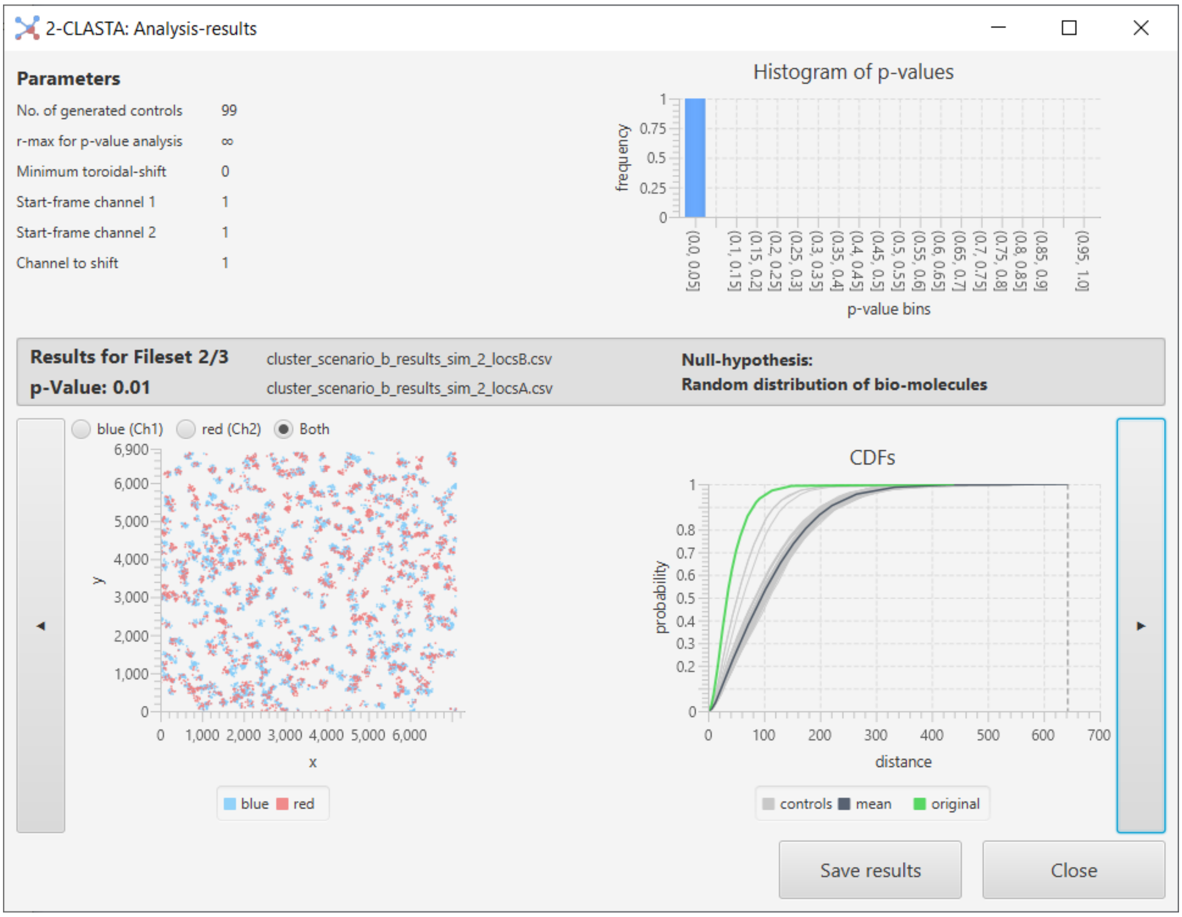

Figure 3: Results-window for an analysis of three filesets. The parameters and histogram of p-values apply for all filesets. The lower section shows selected regions of interest and cumulative distribution functions of the analyzed data for each fileset.

For convenience, the chosen **parameters** are indicated. The histogram shows all obtained p-values.

The user can further display the single molecule localization map, the CDFs and the obtained p-value for each analyzed fileset. In the CDFs, light green indicates the nearest-neighbor distribution of the input data, light gray and black the distributions of the transformed data and its mean, respectively. The vertical-buttons on each side allow users to browse between the filesets.

Export

The **Save results**-button prompts for a filename (without file-extension) and then exports the results in two separate files:

- **Html:** A comprehensive Html-report on the analysis, including the chosen parameters, all rendered charts, the selected regions of interest and the resulting p-values. The report is packaged into a single Html-file, i.e. the file contains all images and style-information, in order to ensure easy exchange with others.
- **Csv:** A comma-separated file containing one row for each fileset, providing the specified channel-names, filenames, the channel name of the shifted channel, the chosen regions of interest and the resulting p-values.

When the export is completed, the target-folder will be opened and the Html-report will be shown.

The **Close**-button terminates the analysis and deletes all used data from the memory.

References

[1] A. M. Arnold, M. C. Schneider, Christoph Hüsson, Robert Sablatnig, Mario Brameshuber, Florian Baumgart, and Gerhard J. Schütz. Verifying molecular clusters by 2-color localization microscopy and significance testing. 2019. submitted.

[2] H. W. Lotwick and B. W. Silverman. Methods for analysing spatial processes of several types of points. *J. R. Statis. Soc.*, 44:406–413, 1982.
